# Differential Smad2/3 linker phosphorylation is a crosstalk mechanism of Rho/ROCK and canonical TGF-β3 signaling in tenogenic differentiation

**DOI:** 10.1038/s41598-024-60717-z

**Published:** 2024-05-06

**Authors:** Michaela Melzer, Sabine Niebert, Manuela Heimann, Franziska Ullm, Tilo Pompe, Georgios Scheiner-Bobis, Janina Burk

**Affiliations:** 1 Equine Clinic (Surgery, Orthopedics), Faculty of Veterinary Medicine, Justus-Liebig-University, 35392 Giessen, Germany; 2 Institute of Physiology, Pathophysiology and Biophysics, University of Veterinary Medicine Vienna, Vienna, Austria; 3 Institute of Anatomy, Histology and Embryology, Faculty of Veterinary Medicine, Justus-Liebig-University, 35392 Giessen, Germany; 4 Institute of Biochemistry, Faculty of Life Science, Leipzig University, 04103 Leipzig, Germany; 5 FILK Freiberg Institute GmbH, 09599 Freiberg, Germany; 6 Institute of Biochemistry and Endocrinology, Faculty of Veterinary Medicine, Justus-Liebig-University, 35392 Giessen, Germany

**Keywords:** Tenogenic differentiation, Mesenchymal stem cells, Linker phosphorylation, Smad, Mesenchymal stem cells, Stem-cell differentiation

## Abstract

The transforming growth factor (TGF)-β3 is a well-known inducer for tenogenic differentiation, signaling via the Smad2/3 pathway. Furthermore, other factors like extracellular matrix or mechanical force can induce tenogenic differentiation and possibly alter the response to TGF-β3 by signaling via the Rho/ROCK pathway. The aim of this study was to investigate the interplay of Rho/ROCK and TGF-β3/Smad signaling in tenogenic differentiation, with the Smad2/3 molecule hypothesized as a possible interface. Cultured as monolayers or on collagen I matrices, mesenchymal stromal cells (MSC) were treated with the ROCK inhibitor Y-27632 (10 µM), TGF-β3 (10 ng/ml) or both combined. Control cells were cultured accordingly, without Y-27632 and/or without TGF-β3. At different time points, MSC were analyzed by real-time RT-PCR, immunofluorescence, and Western blot. Cultivation of MSC on collagen matrices and ROCK inhibition supported tenogenic differentiation and fostered the effect of TGF-β3. The phosphorylation of the linker region of Smad2 was reduced by cultivation on collagen matrices, but not by ROCK inhibition. The latter, however, led to increased phosphorylation of the linker region of Smad3. In conclusion, collagen matrices and the Rho/ROCK signaling pathway influence the TGF-β3/Smad2/3 pathway by regulating different phosphorylation sites of the Smad linker region.

## Introduction

Tendon injuries are a frequently occurring problem in athletes but also in the general population. Tendons, such as the rotator cuff, Achilles tendon and patellar tendon are predisposed for injuries, as those are often subject to continuous overload. Once injured, tendons have a limited healing capacity due to their hypocellular and hypovascular nature. Therefore, scar formation and calcification often occur during tendon healing. The scar tissue has a lower stretch and load capacity resulting in weak spots at the junction between scar tissue and healthy tendon tissue, thereby increasing the risk of re-injury. Consequently, the focus of regenerative therapies is on improving tendon healing and preventing scarring.

Mesenchymal stromal cells (MSC) cannot only modulate the inflammatory response in tendon injuries, but also produce and remodel new functional tendon tissue. Accordingly, MSC support surrounding cells by releasing soluble factors, as well as contribute to the formation of tendon matrix themselves through tenogenic differentiation, matrix synthesis and remodeling. This effect is of crucial interest for the long-term efficacy of MSC therapies and their use in chronic tendon lesions. When MSC are applied into tendon lesions, they are subjected to many different stimuli exerted by soluble factors as well as matrix components and structures. All these components can affect tenogenic differentiation and thereby alter the treatment outcome. Thus, it is important to have a better understanding of the different stimuli, their signaling pathways and their interaction with each other.

Transforming growth factor (TGF)-β3 plays an important role in tendon healing and is highly expressed in the late phase of remodeling. Additionally, it is a reliable inducer of tenogenic differentiation in MSC and can reduce scar formation during tendon healing^1–3^. After binding to its receptor, TGF-β3 mediates its signal either canonically via the Smad2/3 pathway or non-canonically via several serine/threonine kinases. The Smad2/3 pathway appears to be the main pathway for mediating TGF-β3 signaling in tenogenic differentiation^4^. However, studies have also shown that the effect of TGF-β can be altered by other soluble factors and matrix components^5,6^. The latter, represented by decellularized tendon matrix, also promoted tenogenic differentiation on their own^7,8^. In this context, the Rho/ROCK pathway, typically known for conveying biomechanical stimuli and regulating the cytoskeleton, has been shown to be essential^9^. Inhibition of ROCK resulted in a reduction or failure of tenogenic differentiation induced by scaffold topography or mechanical stretching^9,10^. However, this trend appears to be reversed when biochemical factors are used to induce MSC differentiation^11–13^. In line with this, we have previously shown that ROCK inhibition promotes TGF-β3-induced tenogenic differentiation^13^. The underlying signaling mechanisms for this synergistic effect have been only partially identified so far.

The signaling molecules Smad2 and 3 are a possible interface between the canonical TGF-β and the Rho/ROCK signaling pathway. Smad2 and 3 consist of a highly conserved MH1-domain, a conserved carboxy-terminal region (MH2-domain) and a non-conserved linker region^14^. The carboxy-terminal region has two important phosphorylation sites, the Ser^465^/Ser^467^ in Smad2 and Ser^423^/Ser^425^ in Smad3. The linker region of Smad2/3 has at least four major sites for phosphorylation, the Ser^245^, Ser^250^, Ser^255^ and Thr^220^ in Smad2 and the Ser^204^, Ser^208^, Ser^213^ and Thr^179^ in Smad3^14^. Upon TGF-β3 binding to its receptor, carboxy-terminal phosphorylation activates Smad2/3, whereupon it forms a complex with Smad4 and migrates to the nucleus to modulate gene expression and initiate tenogenic differentiation. Additional phosphorylation in the linker region has an important regulatory effect on the stability, activity, and nuclear transport of Smads^15,16^. The linker region can be phosphorylated by several kinases, such as MAPKs, ERK, JNK, and ROCK^17^.

Such a regulatory linker phosphorylation could explain the effect of ROCK inhibition on TGF-β3-induced tenogenic differentiation. The aim of this study was to further investigate the TGF-β3/Smad2/3 signaling pathway with respect to Smad2/3 phosphorylation during tenogenic differentiation and to determine whether extracellular matrix stimuli and the Rho/ROCK signaling pathway affect the TGF-β3/Smad2/3 signaling axis via linker phosphorylation of Smad2/3.

## Results

### MSC characterization

MSC were tested for their surface marker profile and their potential of trilineage differentiation. MSC from all donors were capable of differentiation into adipocytes, osteoblasts and chondrocytes (Supplementary Fig. 1). MSC were positive for CD29, CD44, CD73, CD90 and CD105 and largely negative for CD14, CD34, CD45, CD79α and HLA-DR.

### Tenogenic differentiation

Tenogenic differentiation was assessed based on gene expression of selected markers, such as collagen I and III (COL1A2 and COL3A1), tenascin-C (TNC) and scleraxis (SCX). Additionally, gene expression of collagen II (COL2A1) and sox9 (SOX9) was analyzed to ensure the absence of chondrogenic differentiation.

**Figure 1.**
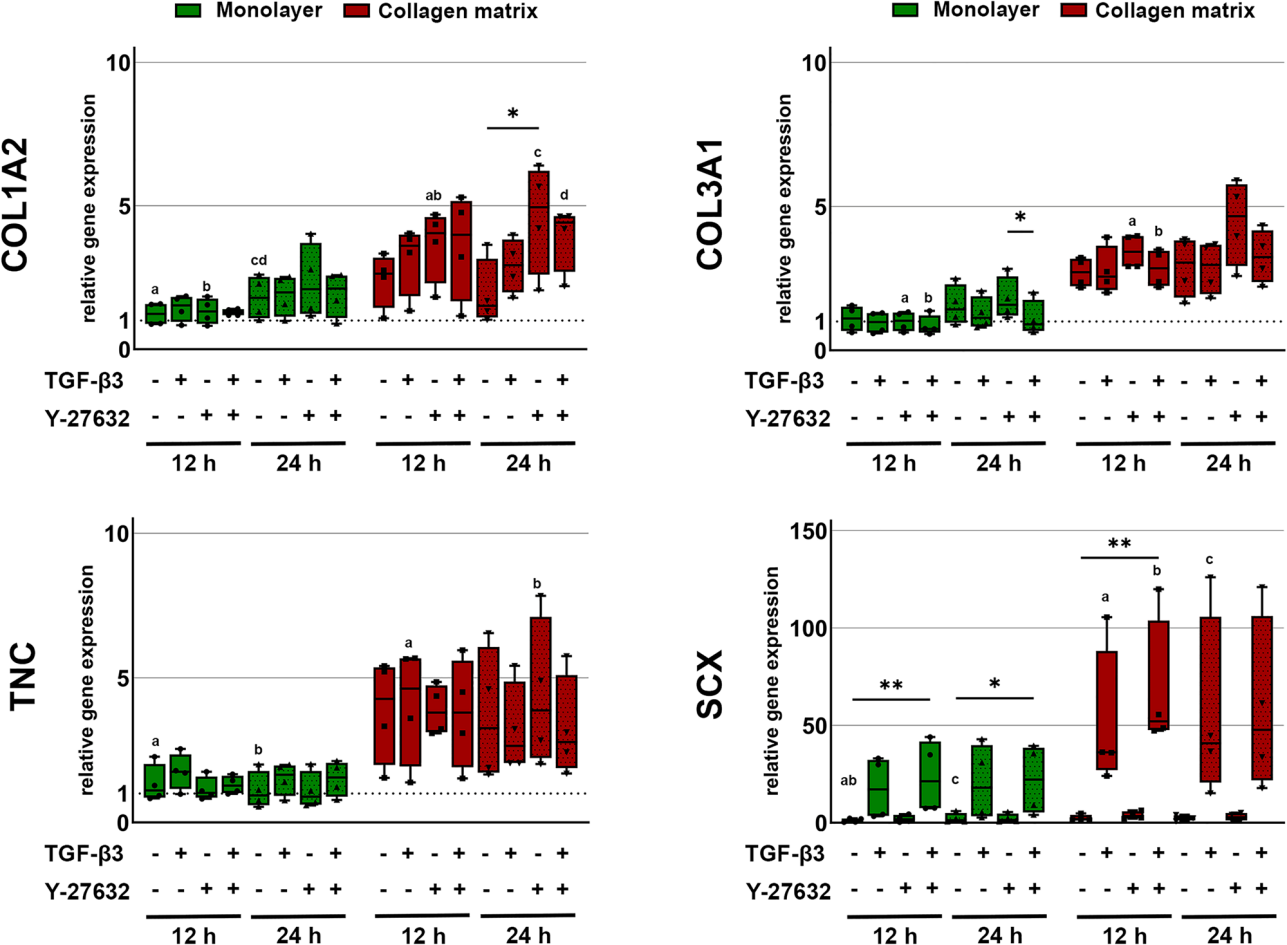
Gene expression of tenogenic markers. Cells cultured as monolayer or on collagen matrix were incubated with 10 ng/ml TGF-β3 and/or 10 µM Y-27632 for 12 h and 24 h. Box plots represent the median values and error bars the minimum to maximum interval. Asterisks mark differences between the stimulation groups with p < 0.05. Letters mark differences with p < 0.05 between collagen matrix and monolayer within same stimulation groups or in comparison to monolayer control (n = 4).

**Figure 2:**
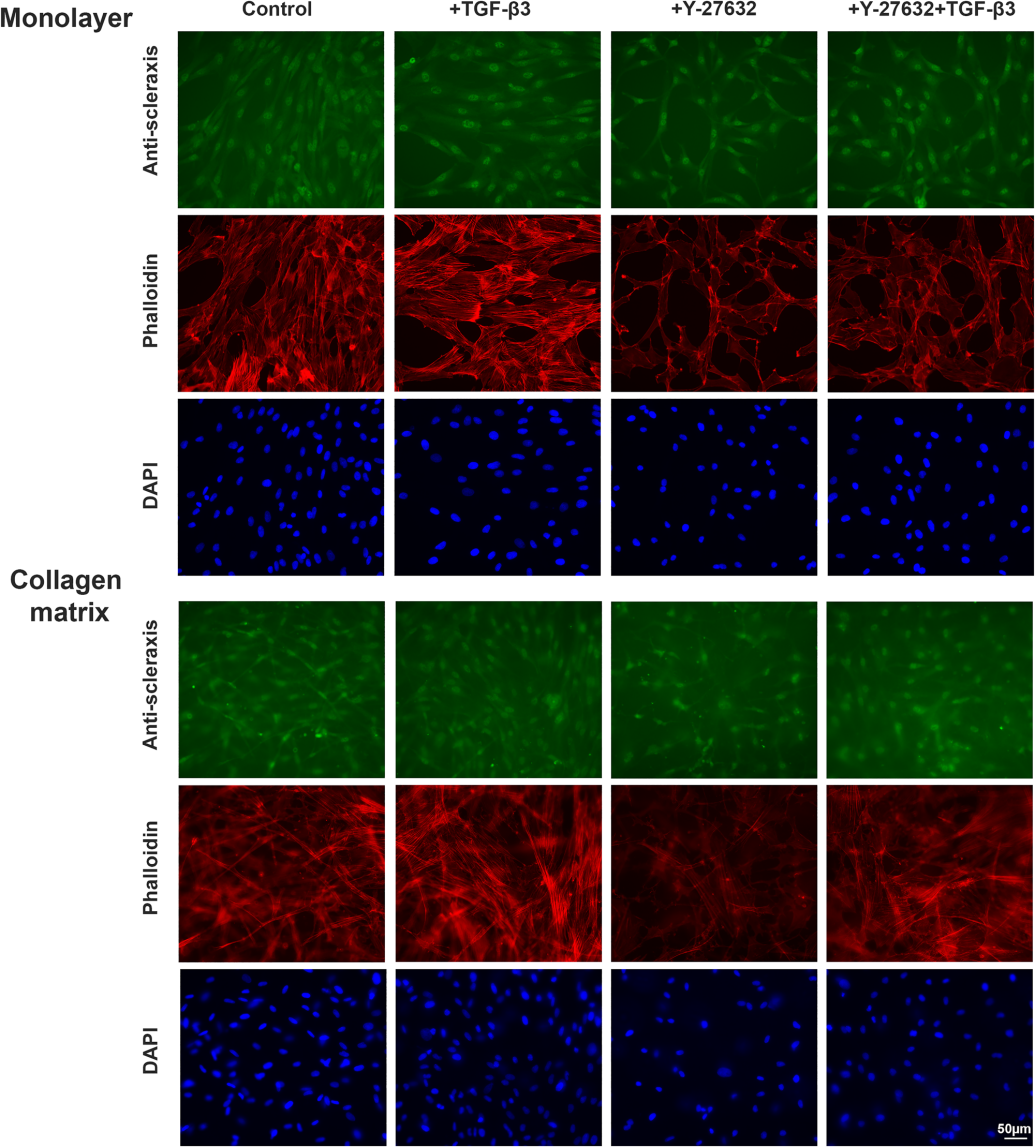
Fluorescence microscopy of MSC as monolayer or on collagen matrix. Cells were incubated with 10 ng/ml TGF-β3 and/or 10 μM Y-27632 and analyzed at 24 h and 72 h. Representative images show scleraxis immunostaining (24 h), phalloidin staining of the actin cytoskeleton and nuclear staining with DAPI (scalebar 50 µm).

Culture of MSC on collagen matrix increased the expression of the extracellular matrix genes COL1A2, COL3A1 and TNC as compared to monolayer culture. While this was not significant between the control groups, the effect was promoted and reached significance between several relevant experimental groups when the ROCK inhibitor Y27623 had been added (p < 0.05). TGF-β3 showed overall less effect on the extracellular matrix gene expression but tended to downregulate COL3A1 (p < 0.05 for monolayer + Y-27632 vs. monolayer + TGF-β3 + Y-27632 after 24 h). However, TGF-β3 strongly upregulated the tenogenic transcription factor SCX, both in the monolayer cultures and the cultures on collagen matrix. This was significant when combining TGF-β3 with ROCK inhibition using Y-27632 (p < 0.01 after 12 h for both monolayer and collagen matrix cultures; p < 0.05 for monolayer culture after 24 h). This upregulation was subject to donor-dependent differences. Two donors moderately upregulated SCX, while the remaining donors showed a strong upregulation. In addition, culture on collagen matrix further promoted SCX upregulation by TGF-β3 and Y-27632 (p < 0.05 compared to the monolayer controls). Data are shown in Fig. 1. Staining of scleraxis on protein level showed that Y-27632 and TGF-β3 were both capable of increasing nuclear translocation of scleraxis (Fig. 2). Inhibition of the Rho/ROCK pathway by Y-27632 was confirmed by a loss of actin fiber organization, resulting in reduced fine drawing in phalloidin staining (Fig. 2).

The transcription factor SOX9 showed a similar regulation as SCX. However, COL2A1 was only expressed at negligible levels as compared to COL1A2 and COL3A1, demonstrating a low risk of chondrogenic differentiation during the period analyzed (Supplementary Fig. 2).

### TGF-β3/Smad axis

To investigate the canonical TGF-β3 signaling pathway in response to TGF-β3, ROCK inhibition with Y-27632 and collagen matrix as support, gene expression of signaling pathway components was analyzed and the different phosphorylation sites of Smad molecules were assessed by Western blotting.

**Figure 3:**
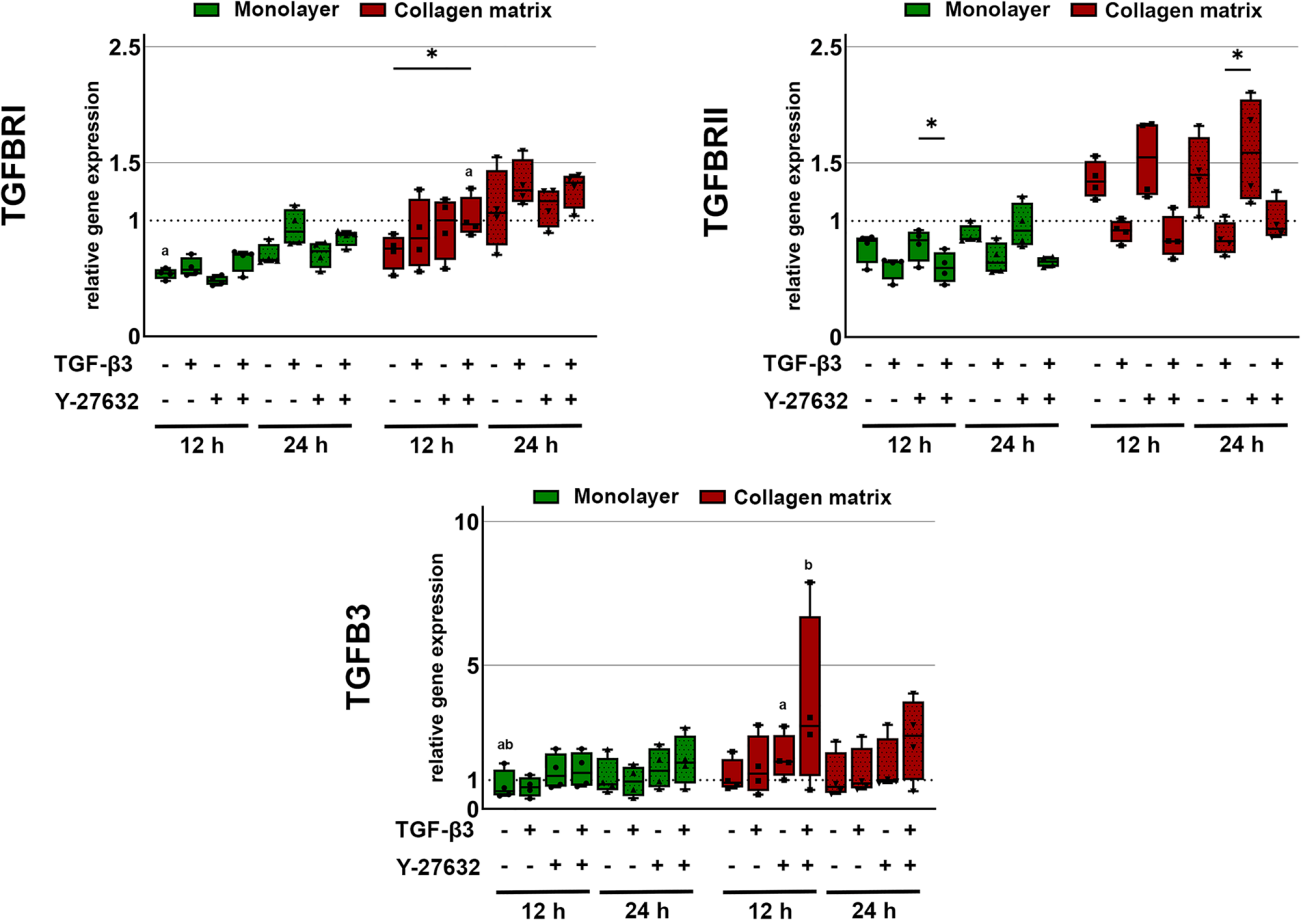
Gene expression of TGFB3 and its receptors. Cells cultured as monolayer or on collagen matrix were incubated with 10 ng/ml TGF-β3 and/or 10 µM Y-27632 for 12 h and 24 h. Box plots represent the median values and error bars the minimum to maximum interval. Asterisks mark differences between the stimulation groups with p < 0.05. Letters mark differences with p < 0.05 between collagen matrix and monolayer within same stimulation groups or in comparison to monolayer control (n = 4).

The presence of TGF-β3 or Y-27632 alone did not alter cellular TGFB3 gene expression. When TGF-β3 and Y-27632 were combined, TGFB3 expression tended to be upregulated, which was enhanced in cultures on collagen matrix (p < 0.01 for collagen matrix culture with Y-27632 or TGF-β3 + Y-27632 vs. monolayer control). The expression of the receptor TGFBRII was higher in the presence of the ROCK inhibitor Y-27632 than with TGF-β3 alone or TGF-β3 + Y-27632 combined (p < 0.01 for TGF-β3 + Y-27632 vs. Y-27632 in monolayer culture and p < 0.01 for TGF-β3 vs. Y-27632 in collagen matrix culture). In this line, TGF-β3 showed a tendency to downregulate TGFBRII, while upregulating TGFBRI (p < 0.05 for TGF-β3 + Y-27632 in collagen matrix culture vs. collagen matrix or monolayer controls). Overall, the changes in TGFBRI and TGFBRII gene expression were rather moderate but supported by culture on collagen matrix (Fig. 3).

**Figure 4:**
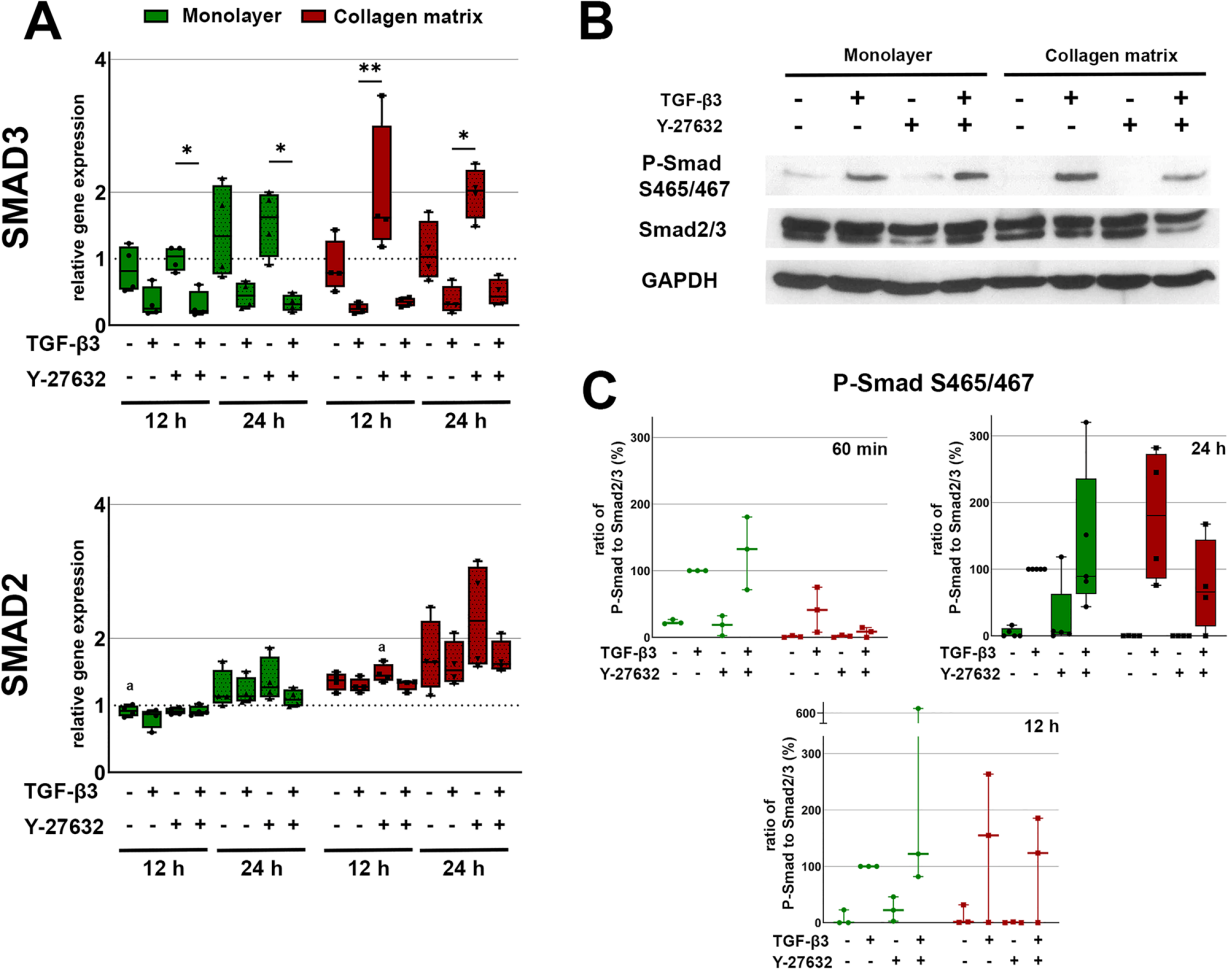
Gene expression and Western blot of Smad2/3. Cells cultured as monolayer or on collagen matrix were incubated with 10 ng/ml TGF-β3 and/or 10 μM Y-27632. (**A**) Box plots represent the relative gene expression of Smad2/3. Asterisks mark differences between the stimulation groups with p < 0.05. Letters mark differences with p < 0.05 between collagen matrix and monolayer within same stimulation groups or in comparison to monolayer control (n = 4). (**B**) Representative western blot result of carboxy-terminal phosphorylation at Ser^465/467^ after 24 h stimulation. (**C**) Quantitative analysis of carboxy-terminal phosphorylation at Ser^465/467^ at 60 min, 12 h (n = 3) and 24 h (n = 5). Box plots represent the ratio of P-Smad to Smad2/3 and dots the values of the different donors.

Gene expression analysis of Smad molecules revealed an upregulation of SMAD3 and SMAD2 expression in the presence of Y-27632, particularly in collagen matrix cultures. For SMAD2, differences were moderate and only significant for the Y-27632 group on collagen matrix compared to the monolayer control (p < 0.01 for Y-27632 in collagen matrix culture vs. monolayer control). The situation was different for SMAD3, where an additional downregulation by TGF-β3 was seen, indicating that TGF-β3 is responsible for the regulation of SMAD3 but not SMAD2. The differential regulation of SMAD3 expression by Y-27632 and TGF-β3 resulted in significant differences between the Y-27632 group and the TGF-β3 or TGF-β3 + Y-27632 groups (p < 0.01 for Y-27632 vs. TGF-β3 + Y-27632 in monolayer culture and p < 0.01 for Y-27632 vs. TGF-β3 on collagen matrix) (Fig. 4).

Western blot analysis demonstrated that the canonical TGF-β3 signaling pathway was activated by TGF-β3 in both monolayer and collagen matrix culture, with carboxy-terminal phosphorylation at Ser^465/467^ of Smad2. ROCK inhibition had no major effect on TGF-β3-induced carboxy-terminal phosphorylation (Fig. 4).

**Figure 5:**
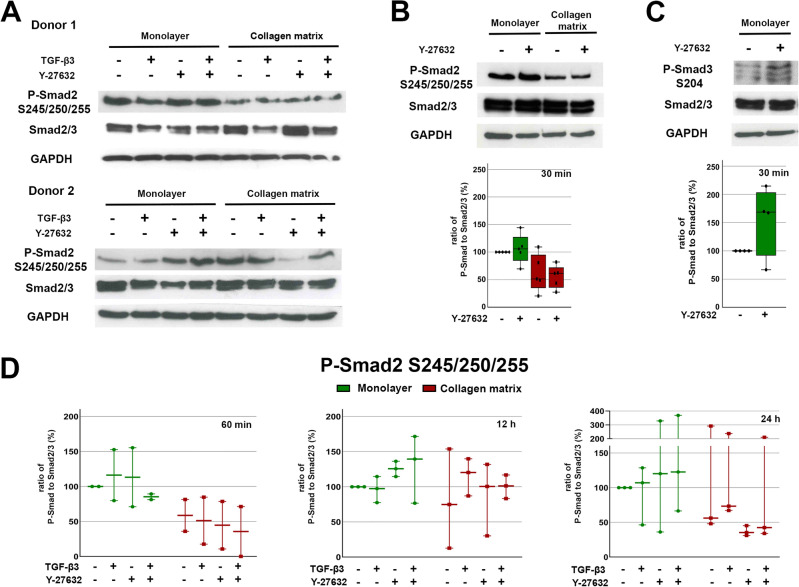
Western blot results of Smad2/3 linker phosphorylation. Cells cultured as monolayer or on collagen matrix were incubated with 10 ng/ml TGF-β3 and/or 10 μM Y-27632. Representative Western blot result of linker phosphorylation at Ser^245/250/255^ of Smad2 after 24 h (**A**) and 30 min (**B**) stimulation and at Ser^204^ of Smad3 after 30 min stimulation (**C**). Quantitative analysis of linker phosphorylation at Ser^245/250/255^ (**B**: n = 5; **D**: n = 3) and Ser^204^ (**C**, n = 4). Box plots represent the ratio of P-Smad to Smad2/3 and dots the values of the different donors.

Examining the linker region (Ser^245/250/255^) phosphorylation of Smad2, there was a reduction in phosphorylation in cultures on collagen matrix compared to monolayer culture. TGF-β3 appeared to have no effect on linker phosphorylation. In a subset of donors, both after 12 h and 24 h, the Y-27632 and TGF-β3 + Y-27632 groups cultured on collagen matrix showed a reduced Smad2 linker phosphorylation and those cultured as monolayers showed an increased phosphorylation. Smad3 linker phosphorylation at Ser^204^ was also increased by ROCK inhibition with Y-27632 in most donors in monolayer culture (Fig. 5). An additional evaluation of Smad2 linker phosphorylation shortly after ROCK inhibition with Y-27632, before TGF-β3 stimulation, again revealed a reduction of linker phosphorylation in collagen matrix cultures, but with no effect of Y-27632 (Fig. 5).

### Integrin-β1/ROCK axis

**Figure 6:**
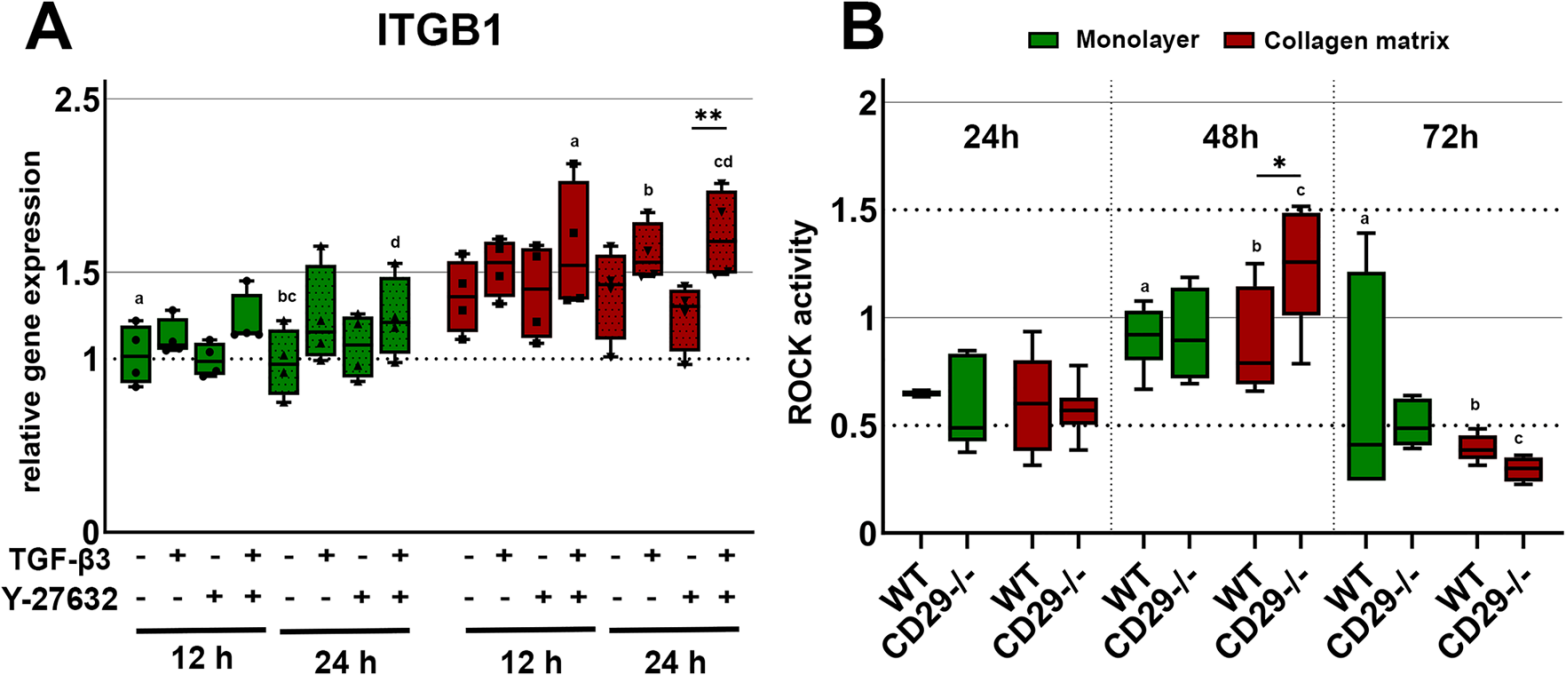
Gene expression of ITGB1 (**A**) and ROCK activity assay (**B**). (**A**) Cells cultured as monolayer or on collagen matrix were incubated with 10 ng/ml TGF-β3 and/ or 10 μM Y-27632. Box plots represent the relative gene expression of ITGB1. Asterisks mark differences between the stimulation groups with p < 0.05. Letters mark differences with p < 0.05 between collagen matrix and monolayer within same stimulation groups or in comparison to monolayer control (n = 4). (**B**) wild type (WT) and CD29^−/−^ cells were cultured as monolayer or on collagen matrix and analyzed after 24, 48 and 72 h. Box plots represents the ROCK activity. Asterisks mark differences between the WT and CD29^−/−^ with p < 0.05 and letters differences between the different timepoints sharing the same letter with p < 0.05 (n = 3).

To investigate whether the CD29/integrin-β1 receptor was involved in modulating ROCK signaling in response to culture on collagen matrix, its gene expression was analyzed for all treatment groups, and a ROCK activity assay was performed in wildtype and integrin-β1^−/−^ cells cultured in monolayers or on collagen matrix (Fig. 6).

Cultivation on collagen matrix alone did not cause a significant upregulation of ITGFB1. However, culture on collagen matrix supported the upregulation of ITGB1 by TGF-β3 and TGF-β3 + Y-27632 (p < 0.01 for TGF-β3 and TGF-β3 + Y-27632 on collagen matrix vs. monolayer control).

In the integrin-β1^−/−^ cells, Western blot and immunofluorescence confirmed the absence of integrin-β1 protein expression (Supplementary Fig. 3). Culturing the wildtype cells on collagen matrix had no measurable effect on ROCK activity compared to monolayer culture. Furthermore, integrin-β1^−/−^ cells showed little difference compared with the wildtype. Only after 48 h of cultivation on collagen matrix, there was a significant increase in ROCK activity in the integrin-β1^−/−^ cells compared with the wildtype (p < 0.05 for integrin-β1^−/−^ vs. wildtype on collagen matrix). However, this increase was transient and no longer detectable after 72 h. After 72 h of cultivation, there was a significant decrease in ROCK activity in all cultures.

## Discussion

Recently, we had shown that ROCK inhibition promotes TGF-β3-induced tenogenic differentiation in MSC, with an increased Smad2/3 nuclear localization^13^ demonstrating activation of canonical TGF-β3 downstream signaling. Previous studies in other contexts already demonstrated cross talk between Rho/ROCK and the TGF-β/Smad pathway and the capability of ROCK to induce phosphorylation of the linker region of Smad molecules, thus regulating their activity^18,19^. The regulatory effect of Smad linker phosphorylation can be inhibitory or reinforcing, depending on the linker phosphorylation site and context^20^. Therefore, here we aimed to explore the signaling crosstalk downstream the tenogenic induction by collagen matrix and TGF-β3, focusing on the contribution of the integrin/Rho/ROCK axis and the Smad2/3 molecules as putative interface integrating activating and inhibiting stimuli.

In this study, both cultivation on collagen matrix and ROCK inhibition supported TGF-β-induced tenogenic differentiation as expected. In addition, ROCK inhibition increased the production of extracellular matrix proteins especially in MSC cultured on collagen matrix. The Smad2/3 molecules were confirmed to play an important role in these processes. Cultivation on collagen matrix reduced Smad2 linker phosphorylation. ROCK inhibition also reduced Smad2 linker phosphorylation, but only in cultures on collagen matrix and with variability between donors, while it increased Smad2 and Smad3 linker phosphorylation in monolayer cultures. TGF-β3 induced carboxy-terminal phosphorylation was consistent between donors and not affected by either component.

The TGF-β and Rho/ROCK signaling pathways play an important role in the regenerative potential of MSC and their adaptation to their extracellular environment. TGF-β is a well-established trigger of tenogenic differentiation and its release is upregulated during tendon healing^21^. The Smad signaling pathway is the canonical transduction pathway downstream of TGF-β receptors, resulting in nuclear translocation of a Smad complex to modulate gene expression^22^. However, MSC act in a highly context-dependent manner and can alter their TGF-β response depending on other environmental stimuli^23^. Therefore, it is necessary to understand the crosstalk between Smad and other, especially mechanosensitive, signaling pathways. Here, Rho/ROCK signaling is of particular interest as it is related to the activation of focal adhesions/integrins by the local ECM environment, thereby mediating mechanical stimuli from the extracellular space^24^. Regarding tendon regeneration, the Rho/ROCK pathway has been considered essential for signal transduction through stretch- and topography-induced tenogenesis^9,10^. Inhibition of Rho/ROCK in human bone marrow-derived MSC subjected to stretching reduced the expression of tenogenic markers and the cells showed a less pronounced tenocyte-like morphology^10^. Contrarily, in the current study, ROCK inhibition promoted TGF-β3-induced tenogenic differentiation. This supportive effect on tenogenic differentiation had already been demonstrated in our previous study in human and equine MSC^13^ and is consistent with other studies that also show a positive effect of ROCK inhibition on paracrine-induced differentiation. In this line, ROCK inhibition promoted the differentiation of MSC into keratinocyte-like cells and increased SOX9 expression during chondrogenic differentiation^12,25^. This suggests that Rho/ROCK has a differential impact on tenogenic differentiation depending on the context. The increase in SOX9 expression as result of TGF-β3 and Y-27632 combination could indicate a favoring of chondrogenic differentiation as well. However, since collagen 2A1 expression is very low, it would be necessary to further investigate the biological relevance of these results in a suitable experimental setup.

With respect to the mechanisms of ROCK in the presence of TGF-β, a study in human breast cancer cells showed that Rho/ROCK is able to phosphorylate the linker region of Smad3 and thereby alter the effect of TGF-β without affecting carboxy-terminal phosphorylation. Specifically, in the breast cancer cells, ROCK inhibition decreased S204 linker phosphorylation^19^. In contrast, we observed an increase in Smad3 S204 linker phosphorylation upon ROCK inhibition in monolayer cultures. In this line, data from a study in mesenchymal cells demonstrated that phosphorylation of S204 is critical for Smad3-mediated COL1A2 promoter activity^26^. Therefore, this particular linker phosphorylation could explain the promoting effect of ROCK inhibition on TGF-β action, as well as the tenogenic effect that the inhibitor alone exerts on matrix protein gene expression.

The key role of Smad3 in tenogenesis was already demonstrated in previous studies with Smad3^−/−^ mice models. Smad3 was found to bind to mohawk and scleraxis, thereby presumably influencing differentiation^27^. The Smad3^−/−^ mice exhibited reduced embryonic tendon formation, resulting in poorer fiber structure and lower tendon loading capacity compared to wild type mice^27^. In contrast, another study associated Smad3 signaling with fibrosis and demonstrated reduced scarring during tendon healing in Smad3^−/−^ mice, yet at the expense of reduced tendon healing^28^. Similarly, different results were observed with other differentiation lineages. During chondrocyte differentiation, Smad3 binds to Sox9 to promote chondrogenic gene expression^29^ and in bone Smad3 binds the osteogenic transcription factor Runx2 to cause TGF-β-mediated inhibition of osteoblast differentiation^30^. The influence on Smad3 linker phosphorylation by combination of ROCK inhibition and TGF-β3 stimulation or cultivation on collagen matrix were not investigated here. The latter, as well as other linker phosphorylation sites of Smad3 should be investigated in future studies to better understand the relevance of Smad3 in the crosstalk between ROCK and TGF-β.

Regarding the effect of collagen matrix, Smad2 linker phosphorylation could be an additional switch, as it was overall reduced in collagen cultures. In contrast to the reinforcing Smad3 linker phosphorylation discussed above, Smad2 linker phosphorylation rather exerts inhibitory effects, which would then be diminished with less phosphorylation. The use of collagen I matrix as support is common practice as over 90% of the tendon matrix consists of collagen I. In addition, collagen I gels are well-established for promoting differentiation and thus allow comparability to previous results. The positive effect of collagen gels has been repeatedly proven^31,32^ and their effect could be further improved by aligning the collagen fibrils^33^. A similar synergistic effect has been described for embryonic stem cells when combining collagen and TGF-β^34^. Integrin-β1 is an important mediator for binding to collagen I and can regulate the Rho/ROCK signaling pathway through its integrin-linked kinase^35^. Previous studies demonstrated an increase in ROCK activity on a 3D collagen matrix^36^. In tumor cells, this regulation was shown to be integrin-β1 dependent^37^. Yet contrary to expectations, no increase of ROCK activity by cultivation on collagen matrix or decrease by integrin-β1 knockout could be detected in the current study. These results suggest that collagen does not or only partially mediate its tenogenic effect via the integrin-β1/Rho/ROCK pathway. However, the different stiffness of the respective cell culture substrates will likely have influenced the activation of other signaling cascades. Tissue culture plastics have a much higher stiffness of 1 × 10^7^ kPa in comparison to collagen matrices, which can be reconstituted in the range of 10 Pa to 50 kPa^38–40^. On stiff scaffolds, MSCs showed reduced tenogenic differentiation and increased activation of the FAK-ERK1/2 signaling pathway compared to softer scaffolds^41,42^. The former could be significantly improved by inhibiting ERK1/2^42,43^. Consequently, low stiffness, like collagen matrices used in this study, promotes tenogenic differentiation by reducing the activity of the ERK1/2 pathway, which is able to regulate phosphorylation of the linker region of Smad2^44,45^. In parallel, ROCK activity is also increased with increasing stiffness and has positive feedback on ERK1/2. While on soft matrix, ROCK inhibition led to a complete inactivation of ERK1/2, on stiff matrix, there was only a slight reduction^46^, indicating a stiffness-related interaction of both pathways. These regulatory differences would explain the different regulation of linker phosphorylation by ROCK inhibition in monolayers and on collagen matrix.

In summary, cultivation on collagen matrix and ROCK inhibition promote TGF-β-induced tenogenic differentiation in human MSC, and our results support the hypothesis that differential linker phosphorylation of Smad2/3 molecules represent the underlying mechanisms. These findings provide a deeper insight into the interplay between the signaling cascades of the extracellular matrix and soluble growth factors. Future studies should shed more light on the contribution of the ERK pathway in the crosstalk of the TGF-β/Smad and Rho/ROCK axes in response to extracellular matrix components.

## Methods

### MSC isolation and characterization

Human adipose-derived mesenchymal stromal cells were isolated from five healthy donors (female, mean ± SD age, 29.6 ± 4.9 years). The subcutaneous adipose tissue was obtained during cosmetic surgeries. All samples were collected after informed consent of the donors and approval by the ethics committee of the Medical Faculty, University of Leipzig (096/17 − ek). All procedures were performed in accordance with relevant guidelines. Isolation was performed as described previously^47^. In brief, the adipose tissue was minced and digested with 0.8 mg/ml collagenase I (Gibco®, Thermo Fisher Scientific GmbH). The recovered cells were seeded in cell culture flasks, detached when 70% confluence was reached and stored frozen until further use. The MSC were analyzed for their three-lineage differentiation and surface marker expression as recommended by the International Society for Cell and Gene Therapy^48^. Surface antigen staining and flow cytometry were performed as previously described (Supplementary Table 1)^47^. Differentiation was performed using StemPro™ differentiation kits (Thermo Fisher Scientific GmbH) and qualitatively assessed using standard stains (Oil Red O, von Kossa and Alcian Blue for adipogenic, osteogenic and chondrogenic differentiation, respectively).

### Collagen I matrix reconstitution

Collagen I matrices were reconstituted on 13-, 20-, and 32-mm glass coverslips coated with 0.14% w/w poly(styrene-*alt*-maleic anhydride) (PSMA; MW 30,000 g/mol; Sigma-Aldrich) according to an established protocol^39,40^. Briefly, rat-tail collagen I (Cultrex® rat collagen I, R&D Systems™) was diluted with 0.2 M acetic acid and 250 mM phosphate buffer to a 2.5 mg/ml collagen I working solution (pH 7.5). All reagents were handled on ice. Total volumes of 40 µl, 95 µl and 400 µl of collagen I solution were applied to the coverslips and the coated coverslips were incubated at 37 °C and humidity for 75 min to complete fibril formation, resulting in collagen matrix layers of approx. 400 µm thickness and roughly 100 Pa elastic modulus. Afterwards, collagen matrices were transferred to well plates and washed twice with phosphate-buffered saline (PBS). Before cell seeding, collagen matrices were equilibrated with the cell culture medium for 1 h.

### Cell culture and stimulation

For experiments, cells were cultured in alpha Minimum Essential Medium (MEM Alpha Medium, Capricorn Scientific GmbH), supplemented with 2.5% human platelet lysate (PL BioScience GmbH), 1% penicillin–streptomycin (Pan-Biotech GmbH), 2 mM l-glutamine (Capricorn Scientific GmbH) and 1 U/ml heparin (PL BioScience GmbH). Cells were seeded at a density of 3000 cells/cm^2^ on plastic culture dishes and at a density of 30,000 cells/cm^2^ on collagen I matrices. After three days of cultivation, cells were stimulated with 10 ng/ml TGF-β3 (R&D Systems®), 10 μM Y-27632 (Tocris), or both. For the combined treatment, cells were first stimulated with Y-27632 for 2 h and then TGF-β3 was added. Cells were stimulated up to 72 h, during which analyses were performed at different time points (30 min, 60 min, 12 h, 24 h, 72 h).

### Western blot

Collagen I matrices were digested with 5 mg/ml collagenase I (Gibco®) for 10 min at 37 °C and monolayer cells were detached with trypsin (Gibco®) for 5 min at 37 °C. Harvested cells were lysed for 5 min at 4 °C in RIPA lysis buffer containing 0.1% SDS, 1% Igepal-CA630, 0.5% Na-Deoxycholate and 1mM EDTA supplemented with proteinase and phosphatase inhibitors (cOmplete™ mini proteinase inhibitor cocktail and PhosStop™, Roche). Protein lysates were then centrifuged for 10 min at 14,000 rpm to remove cell debris. The protein concentration of lysates was determined using a BCA protein assay (Pierce™ rapid gold BCA protein assay, Thermo Fisher Scientific GmbH) using bovine albumin as standard. Prior to use, protein lysates were mixed with loading buffer (Rotiload®, Roth) and denatured at 95 °C for 3 min. The same amount of protein for each sample was loaded on a 10% SDS gel and separated by electrophoresis. Samples were transferred to a PVDF membrane by semi-dry blotting (0.5 V for 30 min). After blocking with 5% milk or BSA, depending on the respective antibody, in a Tris–buffered saline 0.5% Tween (TBST) solution overnight at 4 °C, membranes were stained with antibodies against total-smad 2/3 (1:1000, D7G7, #8685, Cell Signaling Technology), p-smad2 S245/250/255 (1:1000, #3104, Cell Signaling Technology), p-smad S465/467 (1:1000, E8F3R, #18338, Cell Signaling Technology) or p-smad3 S204 (1:500, A24906, Bioworld Technology) for 1 h. Then membranes were washed with TBST three times, incubated with an anti-rabbit HRP-conjugated secondary antibody (1:200, #7074, Cell Signaling Technology) for 1 h and washed again four times. Samples were then visualized using an in-house prepared ECL substrate (0.1 M Tris buffer with 90 mM p-coumaric-acid, 250 mM luminol and H_2_O_2_). The chemiluminescence signal was detected on X-ray films. After stripping of the previously used antibodies with stripping buffer (200 mM glycine pH 2.5, 0.05% Tween 20) two times for 20 min, the membranes were restained with GAPDH antibody (1:8000, A85377) to ensure that equal amounts of protein had been loaded. For quantification, blots were analyzed with GelAnalyzer 19.1. Phospho-smad expression was normalized to total-smad2/3 expression. For a better presentation of the results, the Western blot images were cropped to the relevant sections.

### Gene expression

Gene expression of surface markers, signaling molecules, tenogenic differentiation markers and tendon extracellular matrix components was analyzed by real-time RT-PCR. GAPDH and HPRT1 were used as reference genes. Monolayer cells were directly lysed in RLT buffer supplemented with 1% β-mercaptoethanol. Cells on collagen I matrices were harvested by digestion with 5 mg/ml collagenase I (Gibco®) for 10 min at 37 °C followed by cell lysis. Total RNA was isolated using the RNeasy Mini Kit (Qiagen) including on-column DNase digestion (Qiagen) according to the manufacturer´s instructions. RNA was then converted to cDNA using the RevertAid RT Kit (Thermo Fisher Scientific GmbH). Real-time PCR was performed on the qTower^3^G (Analytik Jena GmbH) Realtime PCR-System using the iQ SYBR Green Supermix (Bio-Rad Laboratories GmbH). 1 µl cDNA (corresponding to 33 ng RNA) was used per reaction. Primer sequences are shown in Table 1. For relative quantification, gene expression ratios were calculated according to the Pfaffl method^49^. The geomean of the housekeeping genes was used as reference gene and an additional monolayer day 0 sample as control for normalization.

**Table 1:** Primer sequences.

Gene	Primer sequence	Accession number	PCR product (bp)
GAPDH	For: CCACTCCTCCACCTTTGACG Rev: CCCTGTTGCTGTAGCCAAATTC	NM_001256799.1	101
HPRT1	For: TGC TGA GGA TTT GGA AAG GGT Rev: GGG CTA CAA TGT GAT GGC CT	NM_000194.3	110
COL1A2	For: CAAGGACAAGAAACACGTCTGG Rev: GTTGGGTAGCCATTTCCTTGG	NM_000089.3	101
COL3A1	For: TGAATATCGAACACGCAAGGC Rev: AAAGCAAACAGGGCCAACG	NM_000090.3	109
COL2A1	For: TCA CGT ACA CTG CCC TGA AG Rev: CGT GAG GTC TTC TGT GAC CG	NM_001844.4	91
TNC	For: CTCTGGAAGACACCGTTGGC Rev: GAAGTGGTGTTTCTTGGAAGCTG	NM_002160.3	101
SCX	For: GCGACGGCGAGAACACC Rev: TCCTTGCTCAACTTTCTCTGGTT	NM_001080514.1	73
SOX9	For: GAG GAA GTC GGT GAA GAA CGG Rev: CCT CTC GCT TCA GGT CAG C	NM_000346.4	233
SMAD3	For: GGTCTGCGTGAATCCCTACC Rev: TTATGTGCTGGGGACATCGG	NM_005902.3	294
SMAD2	For: TCA AGG CAA TTG AAA ACT GCG AA Rev: GTT AGG ATC TCG GTG TGT CGG	NM_005901.6	136
TGFB 3	For: AGCGCTATATCGGTGGCAAG Rev: TCATCCTCATTGTCCACGCC	NM_003239.4	226
TGFBR1	For: ACG TTC GTG GTT CCG TGA G Rev: GAC ACC AAC CAG AGC TGA GTC	NM_004612.4	123
TGFBR2	For: TCC TTC AAG CAG ACC GAT GTC Rev: TTG GAA CCA AAT GGA GGC TCA	NM_001024847.2	110
ITGB1	For: AGT GAA TGG GAA CAA CGA GGT Rev: CAA TTC CAG CAA CCA CAC CAG	NM_002211.4	104

### Immunofluorescence staining

Immunofluorescence staining was performed to examine scleraxis expression and localization under different stimulations. Cells were washed with PBS three times and fixed with 4% formaldehyde (Carl Roth®) for 10 min. Cells were permeabilized with 0.1% Triton X-100 in PBS for 30 min, blocked with 10% donkey serum + 1% BSA in PBS for 30 min and incubated with anti-scleraxis antibody (1:100, ab58655, Abcam) overnight at 4 °C. Subsequently, cells were incubated with donkey anti-rabbit IgG secondary antibody (FITC, 406403, BioLegend) for 1 h. To visualize the nuclei and the cytoskeleton, cells were stained with DAPI (1:1000, Carl Roth®) and Phalloidin (Dylight 554, 13054S, 1:200, Cell Signaling Technology) for 20 min. Between each step, cells were washed with PBS three times for 5 min. Images of stained samples were taken each with the same standardized settings on the Leica DMI6000 B microscope (Leica Microsystems).

### Construction of CD29 knockout cells

MSC from an additional donor (female, 35 years) were used to construct CD29 knockout cells. Cells were cultured in mesenchymal stromal cell growth medium 2 (PromoCell) and 1% penicillin–streptomycin. Targets were designed for the gene sequence of ITGB1 with the webtool chop-chop and cloned into a pSpCas9(BB)-2A-Puro (px459) vector with puromycin and ampicillin resistance. The plasmid was transformed into competent bacteria, expanded and isolated with midi-EF isolation kit (Macherey–Nagel) according to manufacturer’s instructions. In parallel, cells were seeded and cultured until a confluence of 70% to 80% was reached. Then, cells were washed and detached with trypsin. 15 µg of isolated plasmid was transfected into 2 million cells via electroporation using the Invitrogen™ Neon™ Transfection System (Thermo Fisher) according to the manufacturer instruction (1400 V, 20 ms, 2 pulses). The transfected cells were cultured on a 100 mm plate in culture medium without antibiotics for 24 h and another 24 h with 1% penicillin–streptomycin. Then, cells were detached, diluted at a ratio of 1:3 and cultured in culture medium with 2 µg/ml puromycin for 5 days. After further 1–2 weeks of cultivation, clones were isolated and assessed for CD29 expression by immunofluorescence and Western blot. Additionally, the target region of the ITGB1 gene of negatively tested clones was sequenced at Microsynth SeqLab GmbH. The sequenced data was compared to the wild type gene sequence of ITGB1 (NM_002211.4).

### Statistical analysis

Statistical analysis was performed using IBM SPSS Statistics 28 software (IBM Deutschland GmbH, Ehningen, Germany). As data were not normally distributed, non-parametric Friedman tests with Bonferroni-adjusted post-hoc tests were used. Differences were considered significant at p < 0.05. Relative gene expression was evaluated for significance regarding various aspects. Firstly, the different stimulation groups were compared with each other within one culture condition (monolayer or collagen). Significances found here are marked with asterisks in the graphs. Secondly, the stimulation groups on collagen were compared with the correlating stimulation group on monolayer to investigate the effect of culture and finally a comparison was made between monolayer control and the different stimulations on collagen matrix. Graphs were designed with GraphPad Prism 9.4.1 (GraphPad Software, San Diego, US).

### Supplementary Information

Supplementary Information 1.

Supplementary Information 2.

## Data Availability

The original contributions presented in the study are included in the article, further inquiries can be directed to the corresponding author.
