# Supplementary figures and images for: Differential Smad2/3 linker phosphorylation is a crosstalk mechanism of Rho/ROCK and canonical TGF-β3 signaling in tenogenic differentiation
Source: Sci Rep. 2024 May 6;14:10393. doi: 10.1038/s41598-024-60717-z (PMC11074336; doi:10.1038/s41598-024-60717-z)

## Original blots

Figure 3

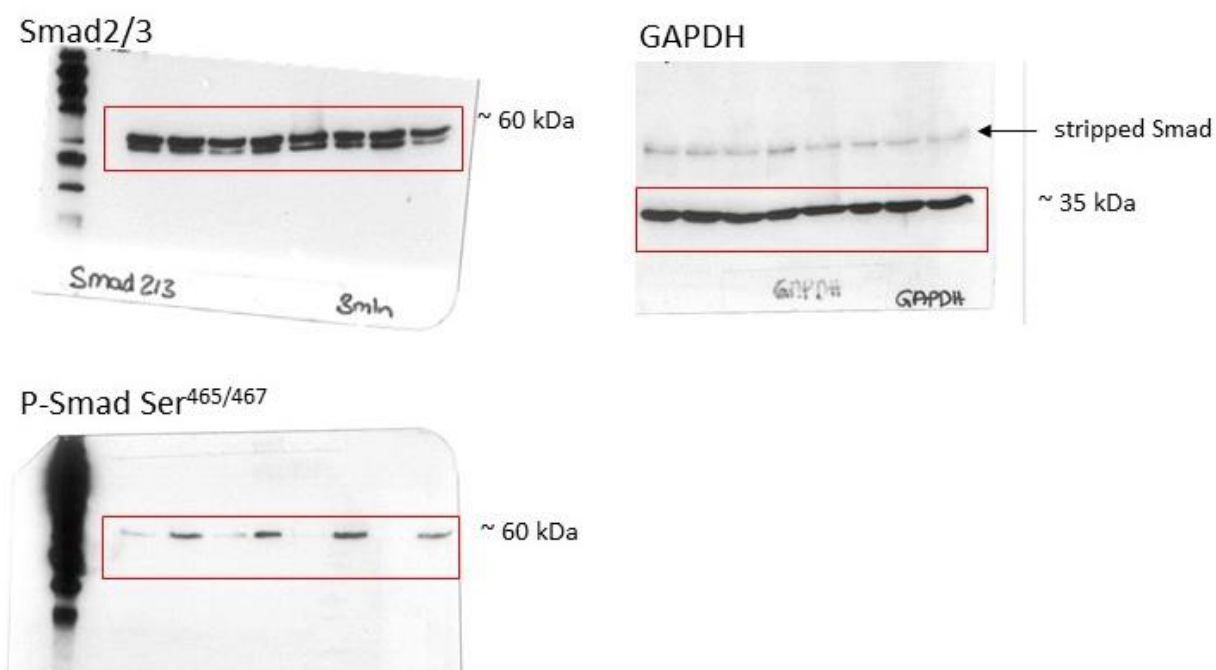

Figure 5

(A)

Donor 1

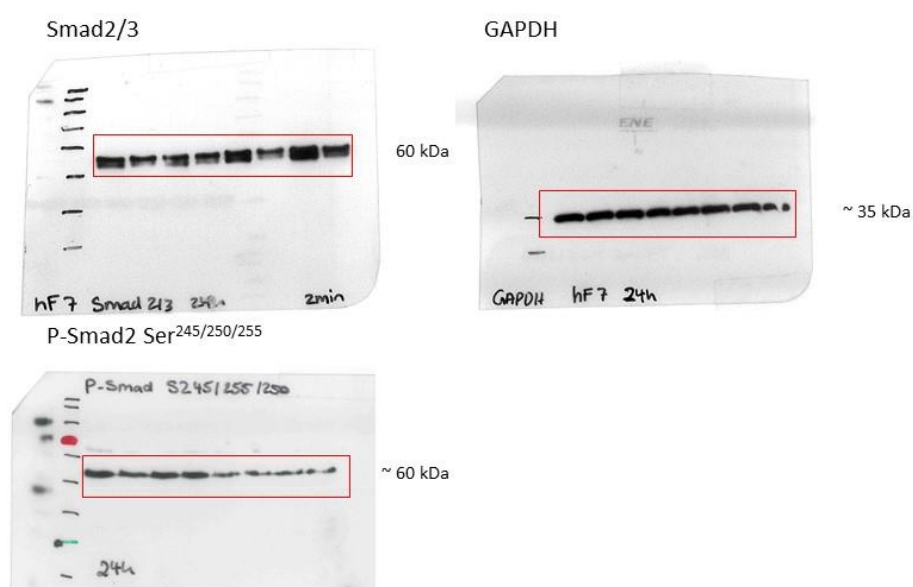

Donor 2

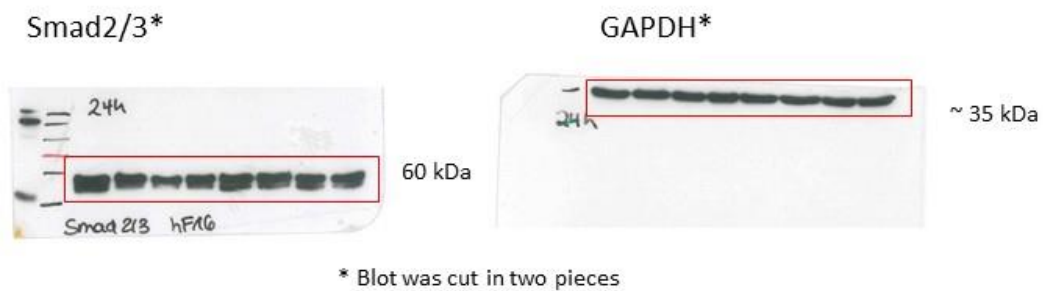

P-Smad2 Ser<sup>245/250/255</sup>

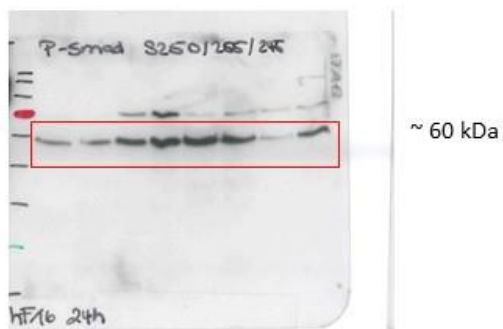

(B)

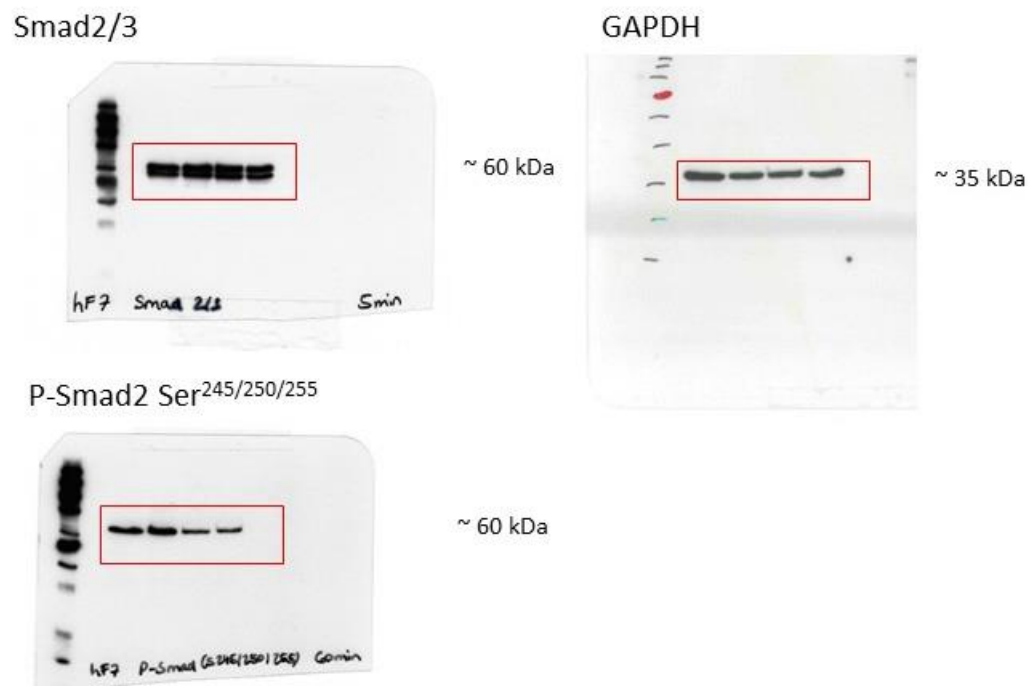

(C)

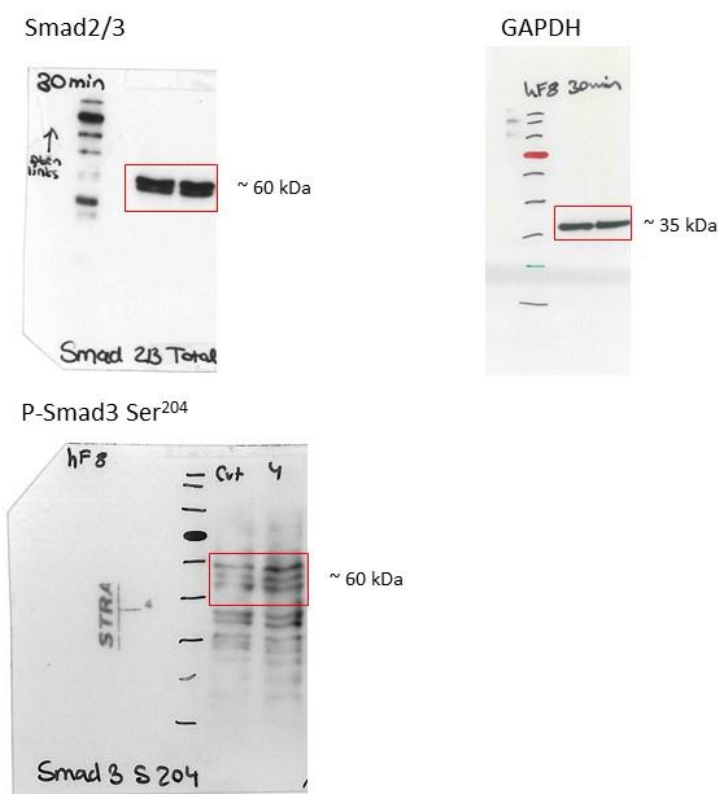

Supplementary Figure 3

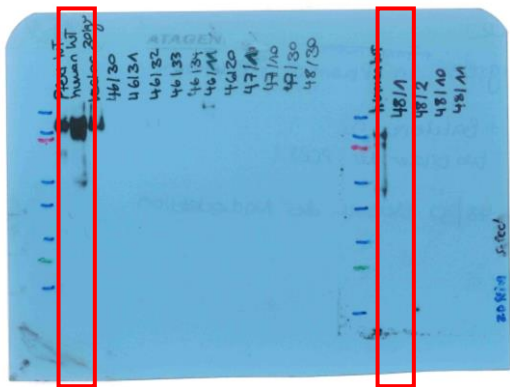

Supplement: Supplementary file 1 — Supplementary Information 1. [file 41598_2024_60717_MOESM1_ESM.pdf]
