# Supplementary material for: Differential Smad2/3 linker phosphorylation is a crosstalk mechanism of Rho/ROCK and canonical TGF-β3 signaling in tenogenic differentiation
Source: Sci Rep. 2024 May 6;14:10393. doi: 10.1038/s41598-024-60717-z (PMC11074336; doi:10.1038/s41598-024-60717-z)
Supplement: Supplementary file 2 — Supplementary Information 2. [file 41598_2024_60717_MOESM2_ESM.pdf]

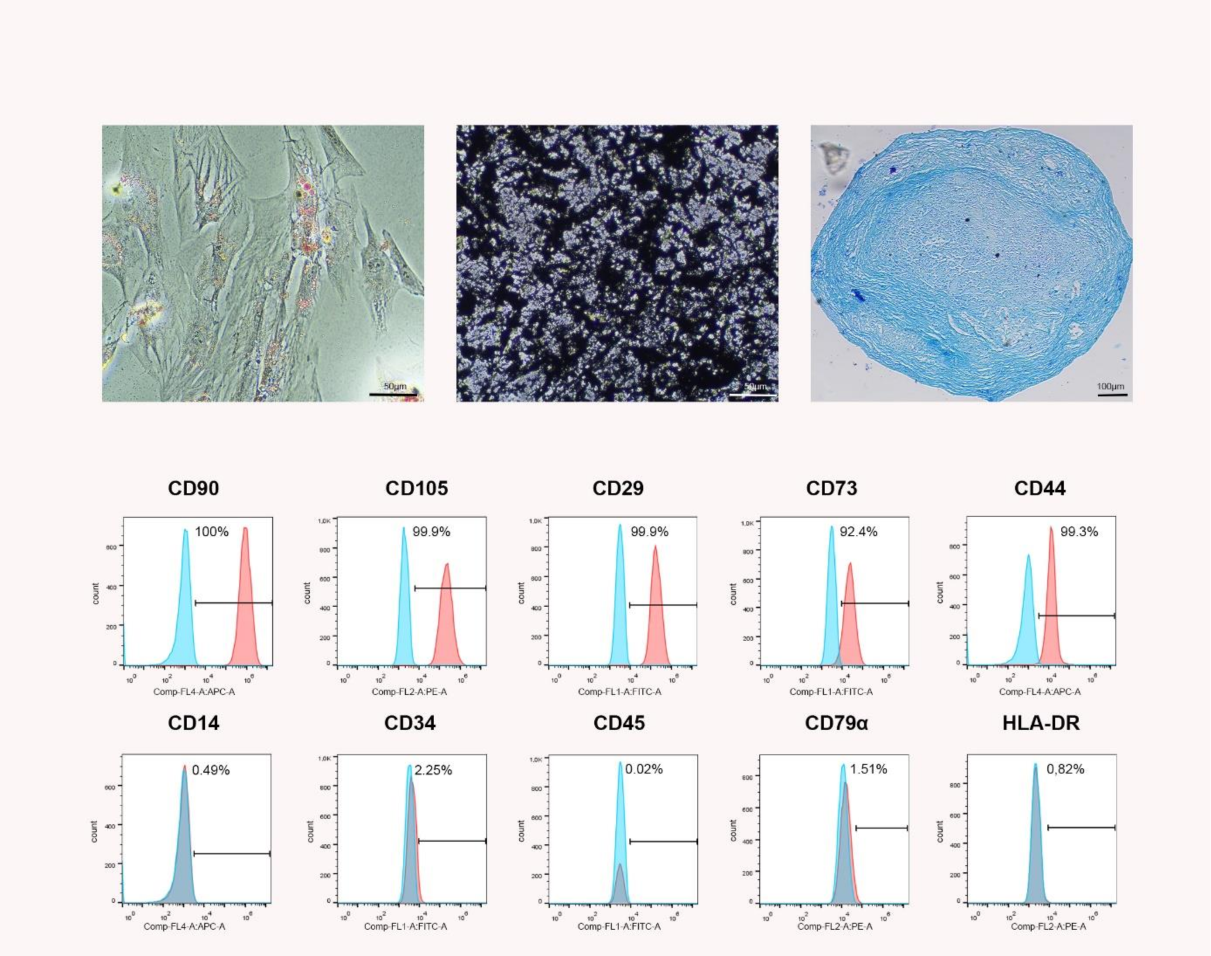

Supplementary Figure 1

Trilineage differentiation and surface marker expression. Representative images show adipogenic, osteogenic and chondrogenic differentiation after oil red O, von Kossa and Alcian blue staining, respectively. The histograms display the surface antigen expression as determined by flow cytometry in a representative sample (blue: isotype control; red: respective antibody staining).

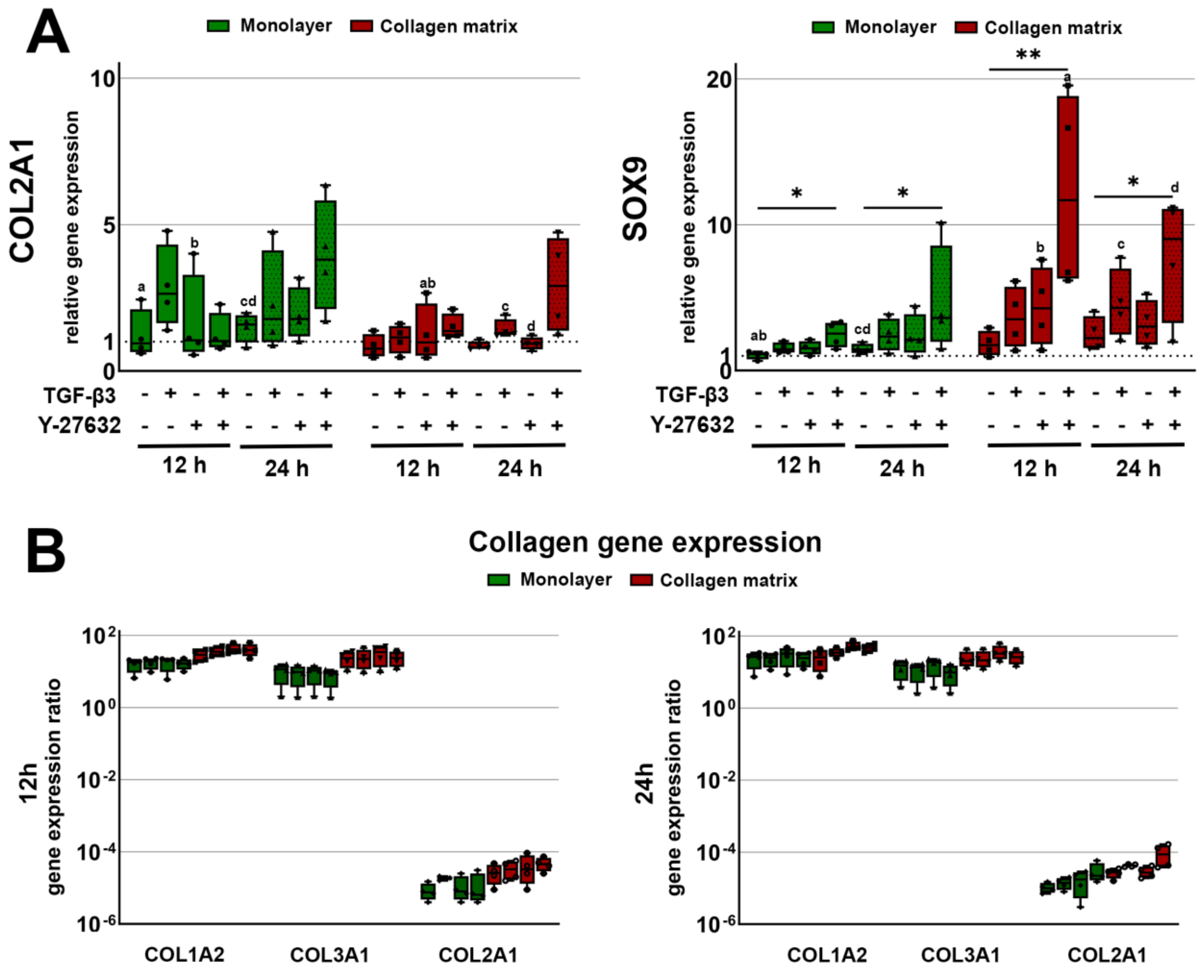

Supplementary Figure 2

Gene expression of the chondrogenic markers COL2A1 and SOX9 (A). Cells cultured on monolayer or collagen matrix were incubated with 10 ng/ml TGF-β3 and/or 10 µM Y-27632 for 12 h and 24 h. Asterisks mark differences between the stimulation groups with $p < 0.05$. Letters mark differences with $p < 0.05$ between collagen matrix and monolayer within same stimulation groups or in comparison to monolayer control (n = 4). (B) Gene expression ratios of COL1A2, COL3A1 and COL2A1, demonstrating the negligible expression levels of COL2A1 as compared to the other collagen types. For the gene expression ratio, the target gene was normalized to the correlating housekeeping genes to allow comparability of the expression levels between the different genes. The groups have the following order control, TGF-β3, Y-27632, TGF-β3 + Y-27632.

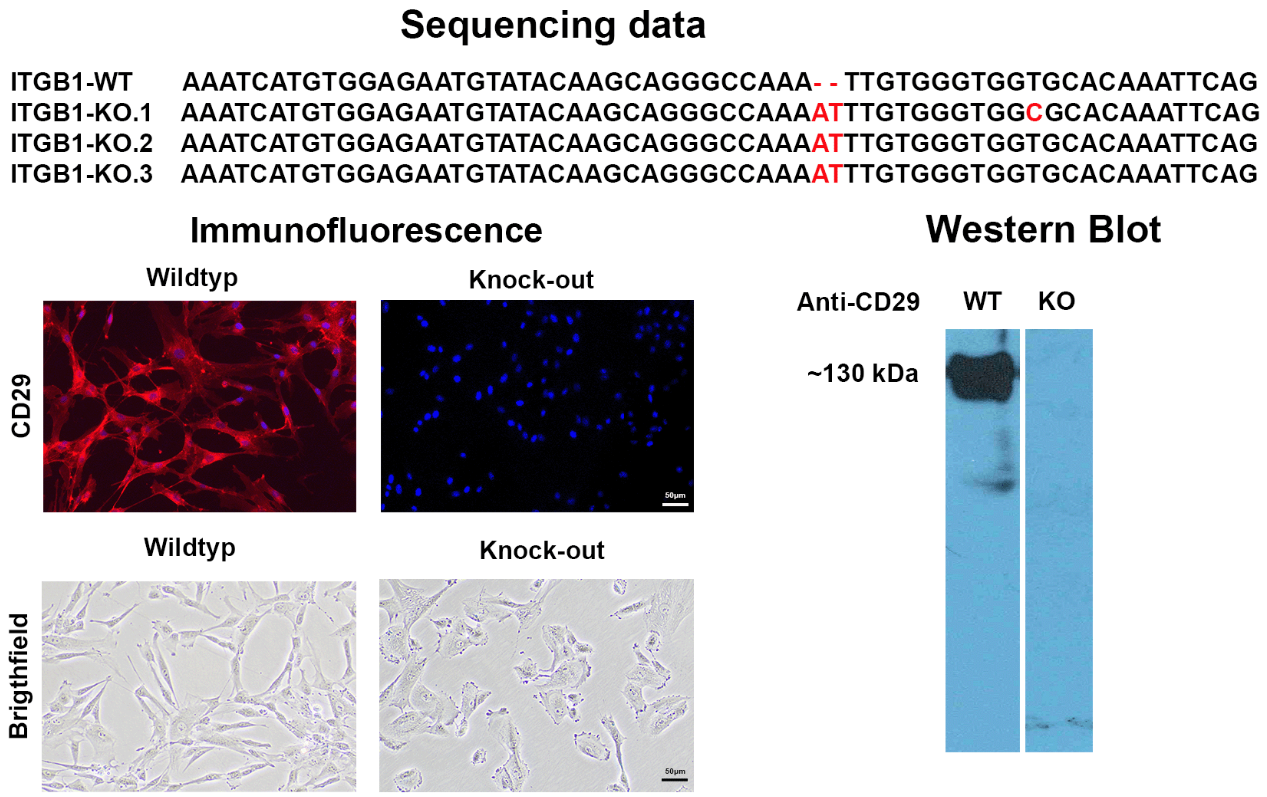

Supplementary Figure 3

CD29$^{-/-}$ knockout cells. The knockout of CD29 was tested by gene sequencing, immunofluorescence and western blot. Representative images and target sequences of the cell clone chosen for the experiments.

Antigen	Clone	Reactivity	Species	Isotype	Company	Dilution	Fluorophore
CD90	5E10	human	mouse	IgG1	BD	1:100	APC
CD105	SN6	human	mouse	IgG1	Serotec	1:50	PE
CD73	606112	human	mouse	IgG2b	R&D	1:40	Alexa488
CD44	IM7	rat	rat	IgG2b	BD	1:400	APC
CD29	TS2/16	human	mouse	IgG1	Biolegend	1:100	Alexa488
CD45	F10-89-4	human	mouse	IgG2a	Serotec	1:400	Alexa488
CD34	43A1	human	mouse	IgG3	Santa Cruz	1:100	FITC
CD14	134620	human	mouse	IgG1	R&D	1:100	APC
CD79α	HM57	human	mouse	IgG1	Serotec	1:10	PE
HLA-DR	L243	human	mouse	IgG2a	Biolegend	1:25	-
	sec. ab	mouse	rabbit		Serotec	1:25	PE

Supplementary Table 1

Monoclonal antibodies used for immunophenotyping.
